# A detailed expression map of the PIN1 auxin transporter in *Arabidopsis thaliana* root

**DOI:** 10.1186/s12870-015-0685-0

**Published:** 2016-01-27

**Authors:** N.A. Omelyanchuk, V.V. Kovrizhnykh, E.A. Oshchepkova, T. Pasternak, K. Palme, V.V. Mironova

**Affiliations:** Institute of Cytology and Genetics SB RAS, Novosibirsk, 630090 Russia; Novosibirsk State University, Novosibirsk, 630090 Russia; Institute of Biology II/Molecular Plant Physiology, Centre for BioSystems Analysis (ZBSA), BIOSS Centre for Biological Signalling Studies, University of Freiburg, Freiburg, 79104 Germany

**Keywords:** PIN1, Polar auxin transport, Root meristem, Hypocotyl, iRoCS Toolbox, Image analysis

## Abstract

**Background:**

Theauxin efflux carrier PIN1 is a key mediator of polar auxin transport in developing plant tissues. This is why factors that are supposed to be involved in auxin distribution are frequently tested in the regulation of *PIN1* expression. As a result, diverse aspects of *PIN1* expression are dispersed across dozens of papers entirely devoted to other specific topics related to the auxin pathway. Integration of these puzzle pieces about *PIN1* expression revealed that, along with a recurring pattern, some features of *PIN1* expression varied from article to article. To determine if this uncertainty is related to the specific foci of articles or has a basis in the variability of *PIN1* gene activity, we performed a comprehensive 3D analysis of *PIN1* expression patterns in *Arabidopsis thaliana* roots.

**Results:**

We provide here a detailed map of *PIN1* expression in the primary root, in the lateral root primordia and at the root-shoot junction. The variability in *PIN1* expression pattern observed in individual roots may occur due to differences in auxin distribution between plants. To simulate this effect, we analysed *PIN1* expression in the roots from wild type seedlings treated with different IAA concentrations and *pin* mutants. Most changes in *PIN1* expression after exogenous IAA treatment and in *pin* mutants were also recorded in wild type but with lower frequency and intensity. Comparative studies of exogenous auxin effects on *PIN1pro*:*GUS* and *PIN1pro*:*PIN1*-*GFP* plants indicated that a positive auxin effect is explicit at the level of *PIN1* promoter activity, whereas the inhibitory effect relates to post-transcriptional regulation.

**Conclusions:**

Our results suggest that the PIN1 expression pattern in the root meristem accurately reflects changes in auxin content. This explains the variability of PIN1 expression in the individual roots and makes PIN1 a good marker for studying root meristem activity.

**Electronic supplementary material:**

The online version of this article (doi:10.1186/s12870-015-0685-0) contains supplementary material, which is available to authorized users.

## Background

The plant hormone auxin affects various processes in plant growth and development [1, 2]. In the root, auxin is the main factor responsible for the formation and maintenance of stem cell niches in the meristem, development of lateral and adventitious roots, and gravitropism among other processes. Auxin regulates many diverse physiological processes due to its uneven distribution in the tissues—an outcome of active transportation mechanisms [3, 4]. PIN efflux carriers, localized in the plasma membrane, are the major contributors to the formation of auxin concentration gradients and maxima [5, 6]. Polar localization of PIN proteins on the plasma membranes creates directed auxin streams in a tissue [7]. For example, in the root meristem, PIN proteins ensure rootward (acropetal) and shootward (basipetal) flows in the vascular system and epidermis, respectively [8].

As compared to other members of the family lack of PIN1 activity results in most severe phenotypes suggesting a crucial role of this protein in auxin transport [7, 9, 10]. The first deviations from normal development are observed in *pin1* mutants at the globular and heart stages of embryogenesis, when one to two thirds of *pin1* embryos show disrupted hypophysis [10]. The basal localization of PIN1 in the plasma membranes provides for directional auxin flow in the globular embryo, where PIN1 in conjunction with other PINs (PIN3, PIN4 and PIN7) contributes to the establishment of the apical-basal embryonic axis. After germination, *PIN1* is expressed in the apical meristems and vascular tissues (reviewed in [2]). Along with pin-shaped inflorescences, fused cotyledons and other shoot abnormalities are evident in *pin1* mutants [9]. The roots of *pin1* seedlings are slightly shortened; their apical meristem is also a bit reduced [7].

In the root, PIN1 mediates rootward auxin flow within the root meristem towards the quiescent centre (QC), which is the site of maximum auxin concentration [3, 7]. PIN1 proteins were predominantly detected on the rootward sides of the stele and endodermis cells along with some expression in the epidermis, cortex and the QC [11, 12]. Additionally, the *PIN1* expression pattern in the root was somewhat variable [12].

In Arabidopsis, the lateral and adventitious roots originate from a founder cell belonging to the protoxylem-pole pericycle in a similar way in roots and hypocotyls, respectively [13–15]. *PIN1* is expressed starting from the first division of the founder cell and, at each division, occupies the newly formed cell boundary. As a result, in the multi-layered primordium, only the outer sides of the peripheral cells do not have PIN1, whereas the inner cells acquire PIN1 at all sides [14]. The preferential positioning of PIN1 towards the lateral root primordium tip became more pronounced at later stages of primordium development. In the hypocotyl, PIN1 expression PIN1 expression visualized with *PIN1pro*:*PIN1*-*GFP* and *PIN1pro*:*GUS* was restricted to the vascular tissue [16].

By directing auxin efflux from cells, PIN1 reduces the cellular auxin concentration. Multiple feedbacks exist in plants to balance this decrease: auxin regulates *PIN1* expression at the levels of transcription, protein stability and subcellular localization [12, 17, 18]. At the tissue level, positive and negative regulation of *PIN1* expression by auxin creates an auxin maximum at a distance from the root end, which may provide for specification and maintenance of the QC [19].

The phenotypic defects in single *pin* mutants are not developed due to ectopic upregulation of the remaining PIN genes, which partially substitute for the activity of the knocked-out gene [20]. In the root, PIN1 functional redundancy was demonstrated in *pin2 single*, *pin2pin3*, *pin2pin4* double and in *pin3pin4pin7*, *pin2pin3pin4* triple mutants [7, 12, 20]. In the *pin2* mutant, *PIN1* was ectopically induced in the *PIN2* expression domain in the cortex and epidermis with polarization, which PIN2 exhibited in these tissues in wild type plants [12].

*PIN1* expression in the Arabidopsis root has been reported in multiple publications, but the data are fragmented and scattered [11, 12, 18, 21–26]. At the same time, testing of *PIN1* expression in the root becomes a pervasive approach in experiments on the regulation of auxin distribution. This requires description of the stable *PIN1* pattern and its possible variations. *PIN1* activity in the root has also been investigated in other plant species [27–31]. In order to obtain a deeper understanding of the role of PINs in plant growth and development, an in-depth description of *PIN1* expression patterns in Arabidopsis will be helpful.

In this study, we conducted a 3D analysis of *PIN1* expression in *A. thaliana* root of wild type and single *pin* mutants using specific antibodies. We show variations in *PIN1* expression and demonstrate that they occurred exclusively at the border of the PIN1 expression domain.

We also determined quantitatively the changes in *PIN1* expression in response to exogenous auxin treatments using two reporter lines (*PIN1pro*:*PIN1*-*GFP* and *PIN1pro*:*GUS*). We found that there is a dependence between the exogenous auxin dose and the changes in the *PIN1* expression level and domain. Significant differences in auxin response observed between *PIN1pro*:*PIN1*-*GFP* and *PIN1pro*:*GUS* plants allowed us to conclude that auxin activates *PIN1* expression at the level of its promoter activity, whereas auxin inhibits it at the post-transcriptional level.

Based on the similarities in *PIN1* expression changes after auxin treatments and in *pin* mutants with the variable part of its expression pattern in control plants, we suggest that the variability in *PIN1* expression may be explained by slight differences in endogenous auxin levels in the individual roots.

## Results

Whole-mount immunolocalization of PIN1 in *Arabidopsis thaliana* roots and hypocotyls was performed with specific antibodies to PIN1 (See Methods). Three-dimensional analysis of PIN1 expression was carried out with the iRoCS Toolbox [32]. PIN1 expression was detected at the root-shoot junction, in developing lateral root primordia and in the tips of primary roots. Stable and variable features in PIN1 expression are described in detail below.

### PIN1 expression in the root tip

**Fig. 1:**
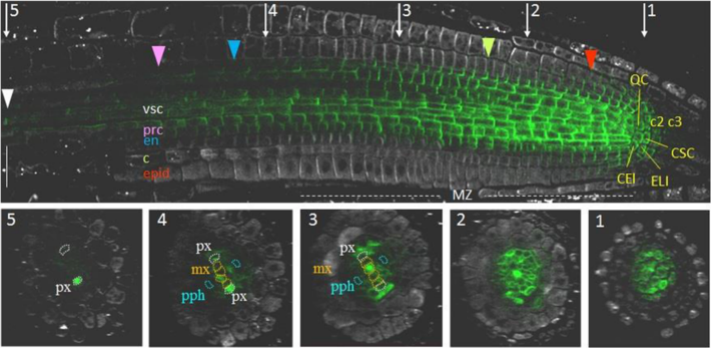
Whole-mount immunolocalisation of PIN1 in *A. thaliana* root tip. A longitudinal section (above) and five transverse sections (1–5) showing anti-PIN1 signal (green channel) and anti-PIN2 staining (white channel). CEI—cortex/endodermis initials, ELI—epidermis/lateral root cap initials, CSC—columella stem cell (columella initials), QC—the quiescent centre, c2—the second columella tier, c3—the third columella tier, epid—epidermis, c—cortex, en—endodermis, prc—pericycle, vsc—vasculature, px—protoxylem, pph—protophloem, mx—metaxylem. Coloured triangles—the end of the expression domain in the respective layer. White triangle—rootward PIN1 location in xylem elements in the elongation zone. MZ—the meristematic zone. Bars = 50 μm

Differentiating stele in the root meristematic zone represent a well-known *PIN1* expression domain [11, 12]. The stele initials and two to four horizontal rows of their descendants had the brightest anti-PIN1 signal in all tested roots (Fig. 1; Additional file 1). In this domain, PIN1 proteins occupied the rootward and lateral surfaces of the plasma membranes, but there was higher accumulation of PIN1 on the rootward sides.

**Fig. 2:**
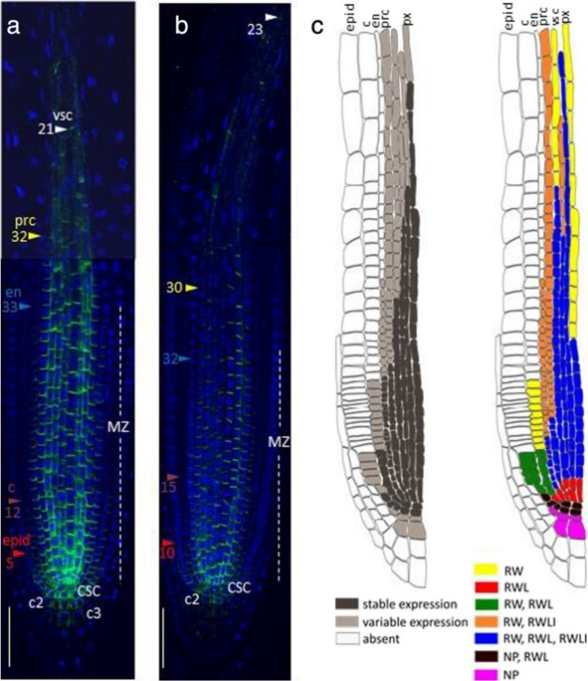
Variability in PIN1 expression domain in different lineages in the root tip. **a** and **b** The primary root tips of two individual 4 dag seedlings labelled with anti-PIN1 (green channel) and DAPI (blue channel). CSC—columella stem cell, c2—the second columella tier, c3- the third columella tier, epid—epidermis, c—cortex, en—endodermis, prc—pericycle, vsc—vasculature, px—protoxylem. Coloured triangles—the end of the expression domain in the respective layer. Bars = 50 μm. **c** A general scheme of stable and variable parts of the PIN1 expression pattern in the root tip of *A. thaliana*. PIN1 expression domain is on the left: dark grey—the stable part of the PIN1 protein domain for the root meristem of a 4 dag seedling, grown on MS medium (see Methods); light grey is its variable part. On the right, stable and variable features of PIN1 polarization are shown. RW—rootward polarity, RWL—rootward and lateral polarity, RWLI—rootward and inner lateral polarity, NP—nonpolar

In differentiating vascular and pericycle cells, the PIN1 signal remained intense and was preferentially positioned rootward, although the amount of signal per cell gradually decreased in the shootward direction (Fig. 1). The decrease in PIN1 expression differed in the cell lineages of the stele. The protophloem and phloem-pole pericycle lost PIN1 signal in the transition domain of the meristem (Fig. 1, Additional file 1; sections 3). A gradual decrease in PIN1 expression as the root protophloem cells differentiated has been shown previously [25]. The procambium and xylem-pole pericycle cells generally lost PIN1 expression at the end of the meristematic zone (Fig. 1, Additional file 1; sections 4). Metaxylem precursor cells and protoxylem lineages expressed PIN1 in the elongation zone. Metaxylem precursor cells lost PIN1 signal just after passing the meristematic/elongation zone border, but expression in the protoxylem was still visible at a distance from the border (Fig. 1, Additional file 1; sections 4; Fig. 2).

In the quiescent center, the first tier of the columella (columella stem cells or initials; CSC), the cortex/endodermis initials and the epidermis/lateral root cap initials, PIN1 proteins occupied the entire plasma membrane. PIN1 signal was not detected in the lateral root cap. It was absent in this lineage immediately after asymmetric division of the epidermis/lateral root cap initials. In the epidermis lineage PIN1 was expressed with the rootward polarity in the first 2–6 cells.

In the first cells of the endodermis lineage, PIN1 proteins occupied the rootward and lateral sides. Further, in endodermal cells, PIN1 proteins remained at the rootward sides and at about one-third of the internal lateral side. The lateral PIN1 disappeared, and the rootward PIN1 became gradually weaker at the beginning of the transition domain. There was no PIN1 expression in the endodermis cells outside the meristematic zone.

In the first cells of the cortex lineage, PIN1 protein occupies both the lateral and rootward sides of the plasma membranes (Fig. 2c). The descendants of these cells exhibit primarily rootward signal, which becomes weaker with each division. In some roots, staining extended to the 16th cortex horizontal row, and various degrees of lateralization were observed (up to the 10th row).

Along with the stable features of the PIN1 expression pattern (e.g., strong expression in the stele), we identified the following variable features differed from root to root: the length of the PIN1 expression domain and the number of cells with the polarization type (rootward, spreading completely or partially localized to the lateral sides) (Fig. 2).

In the stele, PIN1 showed rootward positioning wherever it was expressed, but the presence of the PIN1 protein on the lateral sides of the plasma membranes varied in differentiating stele lineages, starting from the third cell from the QC (Fig. 2c; Additional file 2). PIN1 may occupy both inner and outer lateral sides (as at the beginning of the lineage), or only the inner lateral side, or be absent from the lateral sides. In the pericycle, PIN1 started to switch from equal signal intensities on both lateral sides to decreasing signal on the outer one as early as in the first descendants of the initials. In the middle of the meristematic zone, PIN1 signal usually covered along with the rootward only the inner lateral sides of the pericycle cells. PIN1 disappeared from the lateral sides of the protoxylem and metaxylem cells in the transition domain.

The transition from one polarization type to another occurred in the same cell lineage at variable distances from the QC in different roots. Variability was also detected in the QC, columella initials and their descendants. In some roots, the QC and columella initials did not have polar PIN1. In others, PIN1 was polarized rootward to varying degrees. Rarely, we observed weak non-polar PIN1 signal in the second and third columella tiers. A weak signal was also found using anti-PIN1 antibodies in the second and third columella tiers in *pin1* roots (Additional file 3), suggesting that PIN1 signal there might be non-specific.

### Patterning of PIN1 expression during lateral root development

**Fig. 3:**
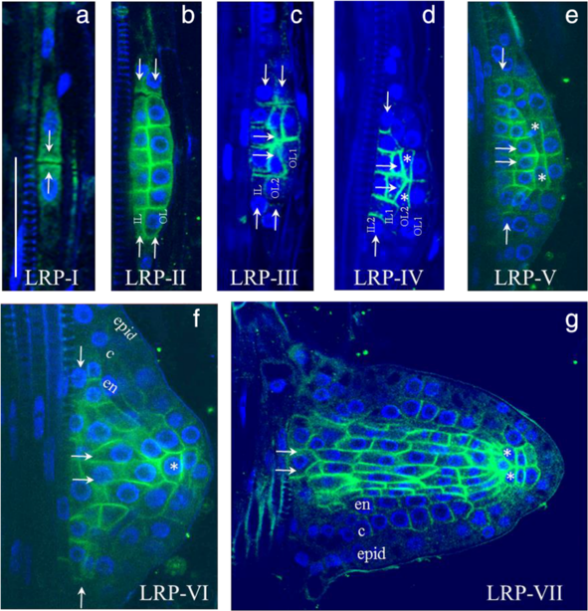
Changes in PIN1 expression during lateral root primordium development. **a**–**g** The seven stages of primordium development are shown in roman numerals. IL (IL1, IL2)—inner layers, OL (OL1, OL2)—outer layers. Pre-specified QC cells are marked by asterisks. Developing tissues: epid—epidermis, c—cortex, en—endodermis. White arrows show the directions of auxin flux. Anti-PIN1 staining is in green, DAPI is in the blue channel. Bars = 50 μm

*PIN1* expression in the pericycle of the maturation zone was observed starting from the first stage of lateral root primordium development (LRP-I), after the first transverse (anticlinal) division of the founder cell. PIN1 localized at the contiguous plasma membranes of adjacent daughters (Fig. 3a). After a series of anticlinal divisions, the LRP-I primordium cells divided periclinally and formed a two-layered LRP-II primordium [33]. At each cell division during LRP-I and LRP-II, PIN1 proteins occupied the contiguous membranes between the daughter cells, marking all newly formed boundaries (Fig. 3b). Cells in the outer layer (OL), followed by the inner layer (IL), undergo periclinal divisions that take the primordium to the LRP-III and LRP-IV stages [33]. At these stages, PIN1 proteins also occupy the membranes between the daughter cells but with a bias in polarity towards the primordium tip (Fig. 3c–d). Cells at the primordium border start differentiating at this time, which can be observed as a decrease (LRP-III) followed by a loss (LRP-IV) of PIN1 expression in this region. These changes likely promote auxin flow towards the primordium tip. From LRP-IV, PIN1 expression gradually decreases in the outer layers (OL2 and OL1) (Fig. 3d, e). Then, QC cells become pre-specified: the WOX5 marker starts to localize to the central cells of OL2 [34]. The pre-specified QC expresses PIN1 without polarization. From LRP-VI, all outer layers—epidermis, cortex and endodermis—can be distinguished (Fig. 3f). As a result, at the LRP-VII stage, the PIN1 expression pattern is similar to the pattern in the primary root: PIN1 is highly expressed in the vasculature and in pericycle precursors, and rootward signal is visible in the developing cortex and endodermis (Fig. 3g).

PIN1 expressing cells were sometimes found close to the developing lateral primordium (Additional file 4). The rootward PIN1 signal was detected in xylem elements (Additional file 4a–d) and individual cells of the outer layers of the primary root near the primordium (Additional file 4e). The PIN1 expression domain in these tissues was dramatically extended after auxin treatment (see below).

### PIN1 expression in hypocotyl

**Fig. 4:**
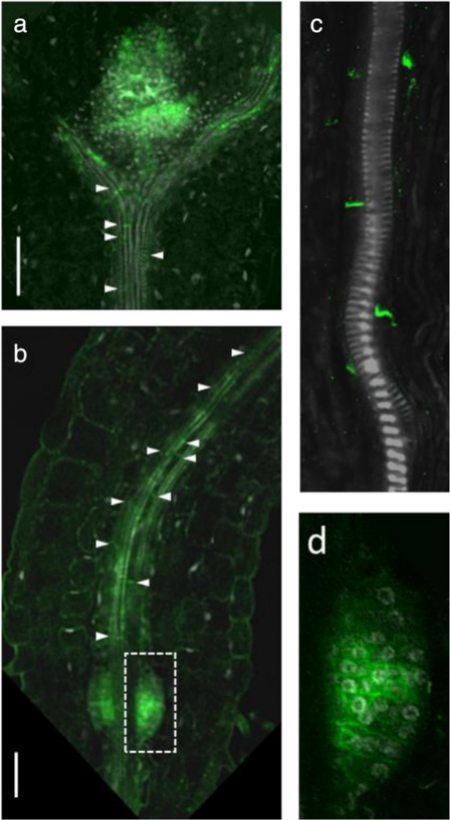
PIN1 expression at the root-shoot junction in 3 dag seedlings. **a** and **b** PIN1 signal (in green) was detected in the vascular elements attached to the mature xylem vessels throughout the whole hypocotyl. PIN1 was polarized rootward (white arrows). **c** The rootward PIN1 signal in the cells flanking the mature xylem vessels of the hypocotyl. **d** Magnified view of the white rectangular region from (**b**). Bars = 50 μm

At the root-shoot junction, PIN1 was detected with rootward polarity in the vascular cells flanking the mature xylem vessels (Fig. 4a–c). In most of the seedlings, within a few days after germination, we also observed PIN1 expression in one or two symmetrical primordia-like organs at the root-shoot border (Fig. 4b). In 3 dag seedlings PIN1 expression in these primordia-like organs resembled those of LRP-V or LRP-VI (Fig. 4d), suggesting that they may give rise to adventitious roots, which in Arabidopsis originate from hypocotyl pericycle cells in a similar way as the lateral roots from root pericycle cells (reviewed in [35]). However, in the older seedlings, we observed that the primordia-like organ lost PIN1 signal and did not develop into adventitious roots. We suggest that these organs are adventitious root primordia that have initiated without developing further. The primordia arrested or delayed in development were previously described for lateral roots [36].

### Exogenous auxin modulates *PIN1* expression

PIN1 expression patterns in wild type plants showed variability in the roots (Fig. 2). This may be caused by variation in the auxin distribution that occurs naturally during plant growth. To test this hypothesis, we assessed the effect of seedling treatments with different concentrations of exogenous auxin (IAA or NAA).

**Fig. 5:**
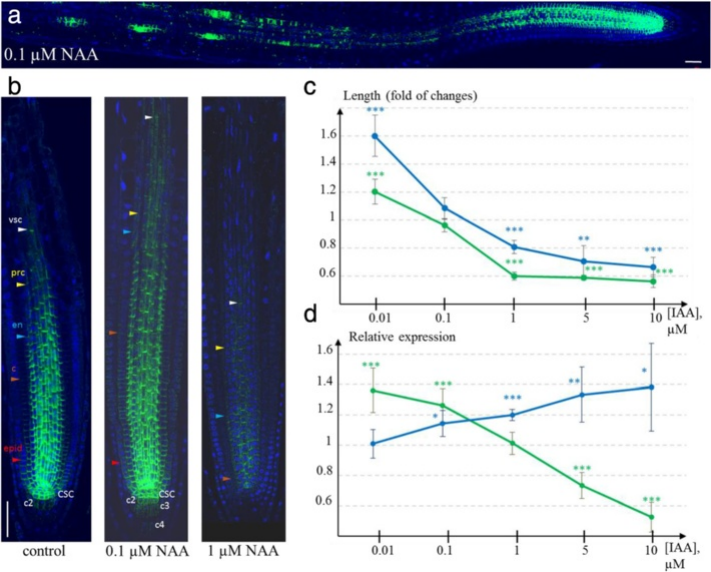
Auxin regulated PIN1 expression in the root tip. **a** and **b** Immunostaining of PIN1 expression in wild type plants treated with low (0.1 μM) and high (1 μM) NAA dosage for 24 h. **a** The effect of exogenous low-level auxin on PIN1 upregulation in the meristem and at the sites of lateral primordia outgrowth. **b** Modulation of PIN1 expression domain under low and high NAA treatments, compared to control. The variation in length of the expression domains for different cell lineages is shown by coloured triangles. CSC—columella stem cell, c2—the second columella tier, c3- the third columella tier, epid—epidermis, c—cortex, en—endodermis, prc—pericycle, vsc—vasculature. Anti-PIN1 staining is in green, DAPI is in the blue channel. Bars = 50 μm. **c** and **d** Quantitative estimates of the changes in PIN1 domain length (**c**) and PIN1 maximal expression intensity (**d**) under exogenous IAA treatments with different dosages. The experimental images were analysed in ImageJ (See Methods). Green line—estimates for *PIN1pro*:*PIN1*-*GFP* plants; blue line—for *PIN1pro*:*GUS* plants. The measured values for each IAA dosage and for each reporter line were normalized to controls. Statistics differences were identified by *t*-test: * *p*-value < 0.05; ** *p*-value < 0.01; *** *p*-value < 0.001

Auxin treatment changed the PIN1 expression domain in the root meristem (Fig. 5a). Auxin effect on PIN1 protein expression in the root apical meristem was dual: auxin upregulated PIN1 expression in low-level treatments (0.01 and 0.1 μM NAA) and inhibited expression at high dosage (1 μM NAA or more) (Fig. 5b). In roots treated with exogenous auxin, we observed both extension (0.01 and 0.1 μM NAA) and shortening (1 μM NAA) of PIN1 expression domains in the vasculature and outer layers—features we noted as variable in control (untreated) plants. For example, in low-level treatments, the PIN1 expression domain was extended in the endodermis, cortex, and stele; in many roots, PIN1 was also detected in the second and third tiers of the columella (Fig. 5b). In contrast, under high-level treatments, the PIN1 domain was significantly reduced in all of the tissues listed above.

To estimate the changes in *PIN1* expression in the primary root meristem quantitatively, we analysed auxin-induced (24 h treatment by 0.01, 0.1, 1, 5 and 10 μМ IAA) reporter activity in *PIN1pro*:*GUS* and *PIN1pro*:*PIN1*-*GFP* plants (see Methods, Additional file 5). Thus, we were able to compare the auxin effect on *PIN1* promoter activity in *PIN1pro*:*GUS* with auxin-dependent post-transcriptional regulation of PIN1-GFP protein in *PIN1pro*:*PIN1*-*GFP*. Using ImageJ [37], we quantified the maximal and average intensities of the reporter signals, as well as the width and length of the PIN1 expression domains in the meristem (See Methods).

Statistical analysis of the measured characteristics supported auxin dose–response of PIN1 expression (Fig. 5c-d). In both lines (*PIN1pro*:*GUS* and *PIN1pro*:*PIN1*-*GFP*), the length of the expression domain in the stele was significantly increased along the root central axis after treatment with low IAA concentration (0.01 μM, *p*-*value* < *0001*) (Fig. 5c). Additionally, the length of the PIN1 expression domain was significantly decreased by high IAA concentrations (above 1 μM) in both lines.

By analysing the maximal intensity of the reporter activity, we found differences between the lines. In the *PIN1pro*:*PIN1*-*GFP* line, a significant increase (by 36 % and 27 %, *p*-*value* < *0.001*) in GFP fluorescence was detected under 0.01 and 0.1 μM IAA (Fig. 5d). After 5 and 10 μM IAA treatment, GFP fluorescence was significantly reduced (by 27 % and 48 %, respectively, *p*-*value* < *0.001*). GUS staining was always increased with increased exogenous auxin (Fig. 5d). Starting from 0.1 μM IAA, the treatment caused significant upregulation of *PIN1* promoter activity (*p*-*value* < *0.05*). Taking into account that the *PIN1pro*:*GUS* line reveals the PIN1 promoter activity by GUS staining and the *PIN1pro*:*PIN1*-*GFP* line monitors the amount of PIN1-GFP protein, we conclude that the auxin effect on PIN1 expression involves both transcriptional and post-transcriptional regulation. Namely, the positive auxin effect on PIN1 expression is explicit at the level of *PIN1* promoter activity, whereas the inhibitory auxin effect relates to post-transcriptional regulation of PIN1 expression.

Summarizing the experimental data on exogenous auxin treatments, we conclude that the variability in the PIN1 expression pattern, which was observed among untreated roots, may be simulated by exogenous auxin treatment. This indicates that differences in endogenous auxin levels among individual roots may be the source of variability in the PIN1 expression pattern.

### PIN1 expression in *pin* mutants

**Fig. 6:**
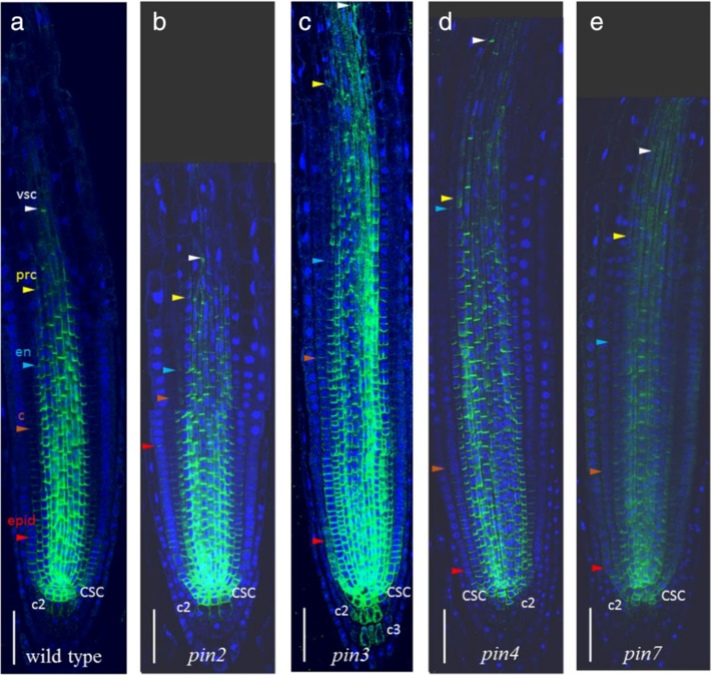
PIN1 expression patterns in pin mutants (**b** – **e**) compared to wild type (**a**). CSC—columella stem cell, c2—the second columella tier, c3—the third columella tier, epid—epidermis, c—cortex, en—endodermis, prc—pericycle, vsc—vasculature. Coloured triangles—the end of the expression domain in the respective layer. Anti-PIN1 staining is in green, DAPI is in the blue channel. Bars = 50 μm

Because PIN proteins mediated auxin efflux from individual cells, we expected that auxin distribution in the root tips of *pin* knockouts would be slightly different from control plants. Indeed, cross-regulation of *PIN1* expression in the *pin2* mutant has been reported before [12]. We analysed the changes in PIN1 immunolocalization in the roots of single knockouts of the genes encoding long PINs (*pin2*, *pin3*, *pin4* and *pin7*). The changes in the PIN1 expression domain compared to controls are summarized in Additional file 6 and Fig. 6.

In wild type plants, PIN1 was expressed and polarized rootward up to the six youngest epidermal cell row (Fig. 2c). In the *pin2* mutant, the PIN1 domain in the epidermis was frequently (75 % of plants) extended up to the twentieth cell row (Fig. 6). In the ectopic domain (from 6th to 20th epidermis rows), the PIN1 protein had shootward polarity inherent to PIN2, which allowed PIN1 to partially substitute the knocked-out gene. In the wild type cortex, PIN1 was expressed rootward, and it gradually declined in intensity up to the 16th cell row from the QC (Fig. 2c). In the cortex of the *pin2* mutant, PIN1 has the same rootward polarization but in an extended domain. We detected a high level of PIN1 expression up to the 18th cell row from the QC, which then gradually decreased moving out towards the 25th cell row. These data support previously reported findings [12]. In addition, in *pin2* mutants, we observed non-polar expression of PIN1 in the second columella tier more frequently than in wild type (85 % of plants). The intensity of the expression was weak but still stronger than in those rare cases when it was detected in this region in wild type.

Changes in PIN1 expression in the vasculature, endodermis and cortex of *pin3*, *pin4*, *pin7* mutants were similar: the PIN1 expression domain extended further to the elongation zone in 60–75 % of plants (Additional file 6; Fig. 6). In the *pin7* mutant, PIN1 also showed ectopic expression in the second columella tier, similar to the *pin2* mutant. In the *pin3* mutant, the domain of PIN1 ectopic expression in the columella was enlarged and covered up to four tiers.

The experimental results show that, in single *pin* mutants, PIN1 expression extended to the sites of normal expression for knocked-out *PIN2*, *PIN3*, *PIN4* and *PIN7* genes. With the exception of the significantly extended PIN1 domain in the epidermis and cortex of *pin2* mutants, all other changes in the PIN1 expression pattern in *pin* mutants were enough close to the spectrum of variability detected in wild type. We assume that the changes in PIN1 expression might be a reaction to changes in endogenous auxin content in *pin* mutants. Tissue-specific auxin accumulation or depletion, which must occur in the absence of one of the PINs, may result in adjustment of the PIN1 expression domain.

## Discussion

Auxin levels control the identities of cells and underlie a wide range of developmental phenomena (reviewed in [1, 2]). Auxin gradients in tissues are the result of auxin movement between cells due to diffusion and active transport. Auxin efflux carriers of the PIN family were shown to be key regulators of auxin distribution (reviewed in [38]). PIN1 is the founding member of this family, and its expression is often used to monitor the effects of other factors on auxin distribution (for example, [21–26]). Various aspects of PIN1 expression but only in relation to the main topics of these papers were described. Here, we present a study focused on PIN1 expression in *A. thaliana* root, which confirmed a number of published facts and revealed some new features of PIN1 expression in the primary root all along its length, up to the root-shoot junction. The regular anatomy of the root apex allowed us to describe the variation and complexity of PIN1 expression in 3D and revealed the stable and varying parts of the expression pattern (Fig. 2, Additional file 2).

The stele is the main PIN1 expression domain in the root meristem [11, 12, 18, 23]. Observations of PIN1 expression in 3D demonstrate that it is not uniform; distinct cell lineages stop expressing PIN1 at different distances from the QC (Fig. 1). The protophloem and protophloem-pole pericycle lost PIN1 signal in the proximal two-thirds of the meristem; procambium and the rest of pericycle lost PIN1 expression from there to the end of the meristem. In contrast, xylem precursors still expressed PIN1 in the distal part of the elongation zone. PIN1 has been suggested to be a marker for non-differentiated cells [25]. Here, we found that it accurately marked the beginning and end of the transition domain in the root tip. As previously described [39], there are no clear markers for the transition domain of the meristem. We suggest that the reference point for the beginning of the transition domain could be the first cell in the protophloem lineage, which lost PIN1 signal. The reference point for the end of the transition domain could be the last protoxylem cell expressing PIN1. Moreover, the end of the PIN1 expression domain in the endodermis coincided with the end of the meristem, which coud be estimated based on the first rapidly elongated cortex cell [40].

In the stele, PIN1 is mainly localized rootward, driving auxin across the vasculature towards the QC. Some spreading of PIN1 protein to the inner lateral sides of the stele cells was observed [18, 23]. In xylem precursors (protoxylem and metaxylem lineages), PIN1 had rootward localization (RW), whereas in procambium cells, PIN1 also occupied the lateral sides [24]. We investigated PIN1 on the rootward and lateral sides of the stele cells in more detail (Fig. 2c). All stele initials and their first daughters have the “procambium mode” of PIN1 expression (RWL) in which PIN1 occupies the rootward and lateral parts of the plasma membrane. Furthermore, there is a difference in PIN1 expression in the descendants of different lineages. Vascular precursors in approximately ten of the horizontal rows from the QC along with stable RW have two types of PIN1 lateral polarization (RWL and RWLI). The RWLI type of polarization means that along with RW, PIN1 protein settles on the inner lateral sides and allows inward auxin flow. The xylem precursors completely lose PIN1 lateral positioning in the upper one-third of the meristematic zone. The other vascular (mainly procambium) cells show variation in the lateral positioning up to the end of the meristematic zone, accompanied by a gradual decrease in PIN1 expression. In the pericycle, constant rootward positioning of PIN1 was also maintained up to the end of the meristematic zone, with the lateral polarity becoming RWLI early in the cell lineage.

Weak rootward PIN1 with some lateral spreading was also reported in the endodermis ([7, 11, 12, 18, 21, 23]. As in the stele initials, in the first cells of the endodermis and cortex lineages, PIN1 is located at the rootward and lateral sides (Fig. 2c). In the endodermis, variations in polarization looked similar to those in the pericycle; along with stable rootward positioning, PIN1 spread to the lower part of the internal lateral side (RWLI).

In the cortex, only weak rootward signal was recorded, mainly in the youngest cells [7, 12, 21, 26]. We additionally showed spreading of PIN1 to the lateral sides in these cells and extension of the PIN1 domain to the middle of the meristematic zone in some roots (Fig. 2c).

Our data on PIN1 expression in the youngest epidermal cells, the QC, the columella initials, the hypocotyl and lateral root primordia (Figs. 1, 3, 4) was consistent with previous reports [12–14, 16, 26].

Comparing our map of PIN1 expression (Fig. 2c) with the auxin distribution map in the root tip [41], we see that stable PIN1 expression occurs in regions with high to intermediate auxin levels. The regions with variable PIN1 expression are characterized by declining auxin. Roots slightly differ in the endogenous auxin level, and this may result in different sizes for the PIN1 expression domain. We hypothesized that changing the auxin level in the root, for example, by auxin treatment, would move the edge between high/intermediate and low auxin levels and this would increase or decrease the length of the PIN1 expression domain. The changes in PIN1 domain length observed after auxin treatment might also correlate with auxin induced changes in the size of the root meristem, as previously described [22, 42, 43].

Indeed, we showed that treatment with low concentrations of auxin (0.01 μM and 0.1 μM NAA) led to lengthening of the expression domain in the stele, endodermis, cortex and columella (Fig. 5a–b). In contrast, treatment with high auxin concentrations (1 μM NAA) reduced the PIN1 expression domain (Fig. 5b). Quantitative analysis of the expression changes in *PIN1pro*:*PIN1*-*GFP* and *PIN1pro*:*GUS* roots after IAA treatment supported the results statistically (Fig. 5c, d).

Similar dose effects have been found previously using another auxin: 0.1 μM 2.4-D was found to be the optimal concentration for upregulation of *PIN1pro*:*PIN1*-*GFP* expression in epidermal cells, whereas PIN1-GFP activity decreased in the stele at higher 2.4-D concentrations [12]. We suggest that the changes in the root auxin level after treatment with exogenous auxin and their influence on the PIN1 expression domain simulated and exaggerated the naturally occurring variations in auxin distribution that occur between individual roots. These changes can influence PIN1 expression.

By analysing the maximal intensities of reporter activity in *PIN1pro*:*PIN1*-*GFP* and *PIN1pro*:*GUS* plants, we found significant differences between the auxin responses of the lines (Fig. 5d). In *PIN1pro*:*PIN1*-*GFP*, GFP fluorescence significantly increased and decreased (*p*-*value* < *0001*) under 0.01–0.1 μM IAA and 5–10 μM IAA, respectively. GUS staining in *PIN1pro*:*GUS* increased with an increase in exogenous auxin dose. These results suggest that positive and negative effects of auxin on *PIN1* are mediated at different levels—transcriptional and post-transcriptional. The shortening of the PIN1 expression domain at a high auxin dosage may be regulated post-transcriptionally.

Finally, we analysed PIN1 expression domain in the single *pin* mutants. We expected that local changes in endogenous auxin level occur in *pin* mutants, which might affect PIN1 expression. PIN2, PIN3, PIN4 and PIN7 are expressed together with PIN1 in the root meristem and may provide redundancy if one of them is mutated [7, 12]. The PIN1 cross-regulation in the *pin2* mutant were described previously [12]. Here, we provide a comparative description of PIN1 expression in single *pin2*, *pin3*, *pin4* and *pin7* mutants (Fig. 6, Additional file 6). In *pin2* and *pin3* mutants, PIN1 almost fully occupies the domain of the knocked-out *PIN* genes. In the case of *pin4*, it is in the vasculature only. In the *pin7* mutant, PIN1 completely occupies the PIN7 expression domain in the vascular system, and it partially occupies the first columella tier. Comparing the expression patterns of PIN1 in *pin* mutants (Additional file 6) with the wild type (Additional file 2), we find that the changes in PIN1 expression mainly fall within the spectrum of variability observed in wild type plants.

By regulating PIN1 expression level and the size of the PIN1 expression domain, auxin determines the efficiency of its own transport. By controlling PIN1 polarity, auxin determines the directions of its own streams. The rootward polarity of PIN1 promotes auxin flow to the root tip. The inner lateral location of PIN1 in the endodermis and pericycle collects auxin from the shootward auxin flow to the vasculature, and PIN1 lateralization in procambial cells allows to accumulate auxin in the narrower stream in xylem precursors. This accumulation is probably important for vascular differentiation inside the meristem [44].

NAA treatments at concentrations as low as 0.1–1 μM (2–4 h) affected PIN1 polarity and caused PIN1 to spread across the entire inner lateral sides in the endodermis and pericycle, but higher NAA concentrations did not show further polarization, suggesting saturation of the effect [18]. This may indicate that the responses of expression level and polarization to auxin are different. In our experiments, comparing the maps of PIN1 expression and its polar locations (Fig. 2c) gave evidence showing that PIN1 polarization may be more sensitive to minor changes in auxin cellular concentrations than the PIN1 expression level.

## Conclusions

PIN proteins reduce the cellular auxin level by carrying auxin out of the cell. As a feedback mechanism, auxin regulates the transcription, stability and polarization of PINs. We have clarified the role of the founding member of the PIN family (PIN1) in these close and interconnected relationships that establish the auxin distribution, which is a key piece of positional information for cell fate determination in the root stem cell niche.

## Methods

### Plant materials

The following plant varieties were used in the experiments: *Arabidopsis thaliana* Col-0 (L.) Heyhn; reporter lines *PIN1pro*:*GUS* (−1388,+82) [kindly provided by Drs Sodnom Sangaev and Alexei Kochetov, Institute of Cytology and Genetics, Novosibirsk, Russia] and *PIN1pro*:*PIN1*-*GFP* [12]; *pin2* (*eir2*-*1*) (CS8058); *pin4*-*3* (NASC: 9368); *pin3*-*5* (NASC: 9364); *pin7*-*2* (NASC: 9366).

### Growth conditions and treatments

Seeds were surface-sterilized and sown on solid Arabidopsis medium (AM; ½ MS medium containing 1 % sucrose, 5 mM MES and 1.1 % agar, pH 5.6 adjusted with KOH). After vernalization for 16 h at 4 °C, seeds were germinated on vertically oriented plates under a 16:8 h light:dark period with a light intensity of 80 μmol s-1 m-2. Four-day-old *pin* mutants were subjected to immunolocalisation.

Three-day-old seedlings (Col-0, PIN1pro:PIN1:GFP and PIN1pro:GUS) grown on the AM medium were transferred to liquid AM supplemented with different concentrations of IAA (0, 0.01, 0.1, 1, 5, 10 μM) or NAA (0, 0.01, 0.1, 0.5, 1 μM) for 24 h.

### Whole-mount in situ immunolocalisation

Immunolocalisation in Arabidopsis plants was performed according to a whole-mount in situ protocol [45]. Briefly, seedlings were fixed in 4 % formaldehyde and treated with methanol. Cell walls were digested with a mixture of Dricelaze and Macerozyme, membranes were permeabilized with the mixture of DMSO and NP40, and the resulting samples were incubated with primary and secondary antibodies. Seedlings were stained with DAPI and mounted on microspore slides with a spacer. Affinity purified primary anti-PIN1 (mouse, clone 7E7F) antibodies were diluted 1:40, anti-PIN2 (guinea pig, clone 192) antibodies were diluted 1:400. The secondary Alexa-488/Alexa 555 conjugated anti-mouse and anti-guinea pig antibodies were diluted 1:400.

### Microscopy

Analysis of the fluorescent signal after *in situ* immunolocalization was performed with a Zeiss Stemi SV11 APO stereomicroscope equipped with a fluorescent HBO lamp and a GFP filter set (488 nm excitation and 530–550 nm emission). Analysis of *PIN1pro*:*PIN1*-*GFP* was performed under an Axio Imager M1 fluorescence microscope. *PIN1pro*:*GUS* plants were treated according to a standard protocol and analysed under a light microscope. For high-resolution images, plants containing fluorescent markers were fixed with 4 % formaldehyde and mounted in Prolong Gold anti-fade reagent containing DAPI (Molecular Probes). Fluorescence was analysed with a Zeiss LSM 5 DUO scanning microscope.

### Image analysis

The confocal images were analysed with the ZEN image browser. Three-dimensional image analysis was performed using the iRoCs toolbox [32]. Quantitative analysis of reporter activity in *PIN1pro*:*PIN1*-*GFP* and *PIN1pro*:*GUS* lines was performed in ImageJ [37]. Images were analysed in the green and red channels for the reporters GFP and GUS, respectively. The maximum and average fluorescence intensity/GUS staining, and the length of the expression domains in stele were measured in individual roots. Image analysis for *PIN1pro*:*GUS* was performed according to a previously described method [46]. The maximal intensities of GFP signal and GUS staining were measured along the central root axis by using the analyse/PlotProfile tool. They were estimated along a thick line that was half the width of the PIN1 domain in the stele. The length of the expression domain corresponded to the distance between the CSC and the closest point along the same thick line with an intensity value below those of the CSC. The significance of the differences in the activities of reporters between the control and auxin treated roots was assessed by *t*-test.

## Additional files

Additional file 1:
**The details of PIN expression in the root tip, visualized in 3D.** The longitudinal section is above. Five transverse section made at different lengths from the QC are shown below. The signal disappears in the upper third of the meristematic zone (MZ) in the protophloem (pph) and protophloem-pole pericycle. In the distal part of elongation zone protoxylem (px), cells still express PIN1. Bars = 50 μm. (TIF 13846 kb) Additional file 2:
**Stable and variable features of the PIN1 expression domain in the meristem.** The first column indicates cell position along the central root axis: CSC—columella initials, CEI—cortex/endodermis initials, ELI—epidermis/lateral root cap initials, QC—the quiescent centre. 1–30 - the cell numbers in the lineage from the QC. RW—rootward polarity, NP—nonpolar, RWL—rootward and lateral polarity (on the both sides of the plasma membrane), RWLI—rootward with spreading to the inner lateral side, RWLIp—the same as RWLI, but only part of the inner lateral side is occupied by PIN1. The typical (most frequent) polarity is highlighted in bold font. (DOCX 15 kb) Additional file 3:
**PIN1 expression pattern in the** ***pin1*** **mutant (negative control).** A weak signal is present in the second and third columella tiers (c2, c3). Anti-PIN1 staining is in green, DAPI is in the blue channel. Bars = 50 μm. (TIF 10907 kb) Additional file 4:
**PIN1 expression in closer proximity to the developing lateral primordium.** a-d. PIN1 signal (red arrows) was detected in the xylem elements. c. Magnification of the white box from figure b. e. PIN1 signal (red arrows) was detected in individual cells of the outer layers of the primary root. The white inset shows the same primordium, but with a different focal plane. Anti-PIN1 staining is in green, DAPI is in the white channel. Bars = 50 μm. (TIF 16686 kb) Additional file 5:
**Auxin treatment effect on the reporter lines PIN1pro:PIN1-GFP and PIN1pro:GUS.** Examples of PIN1-GFP expression (a) and GUS staining (b) are given for the roots under low (0.01 μМ IAA) and high (10 μМ IAA) treatments, and these are compared with the control. Bars = 50 μm. (TIF 7755 kb) Additional file 6:
**PIN1 expression in the roots of** ***A. thaliana pin*** **mutants.** PIN1 positioning on the plasma membrane was described as follows: RW—rootward, RWLI—rootward and lateral internal, SW—shootward. The percentages of roots having the listed features are shown in brackets. QC—the quiescent centre, CSC—columella stem cells, LRC—the lateral root cap. (DOCX 13 kb)
