# Supplementary figures and images for: A detailed expression map of the PIN1 auxin transporter in Arabidopsis thaliana root
Source: BMC Plant Biol. 2016 Jan 27;16(Suppl 1):5. doi: 10.1186/s12870-015-0685-0 (PMC4895256; doi:10.1186/s12870-015-0685-0)

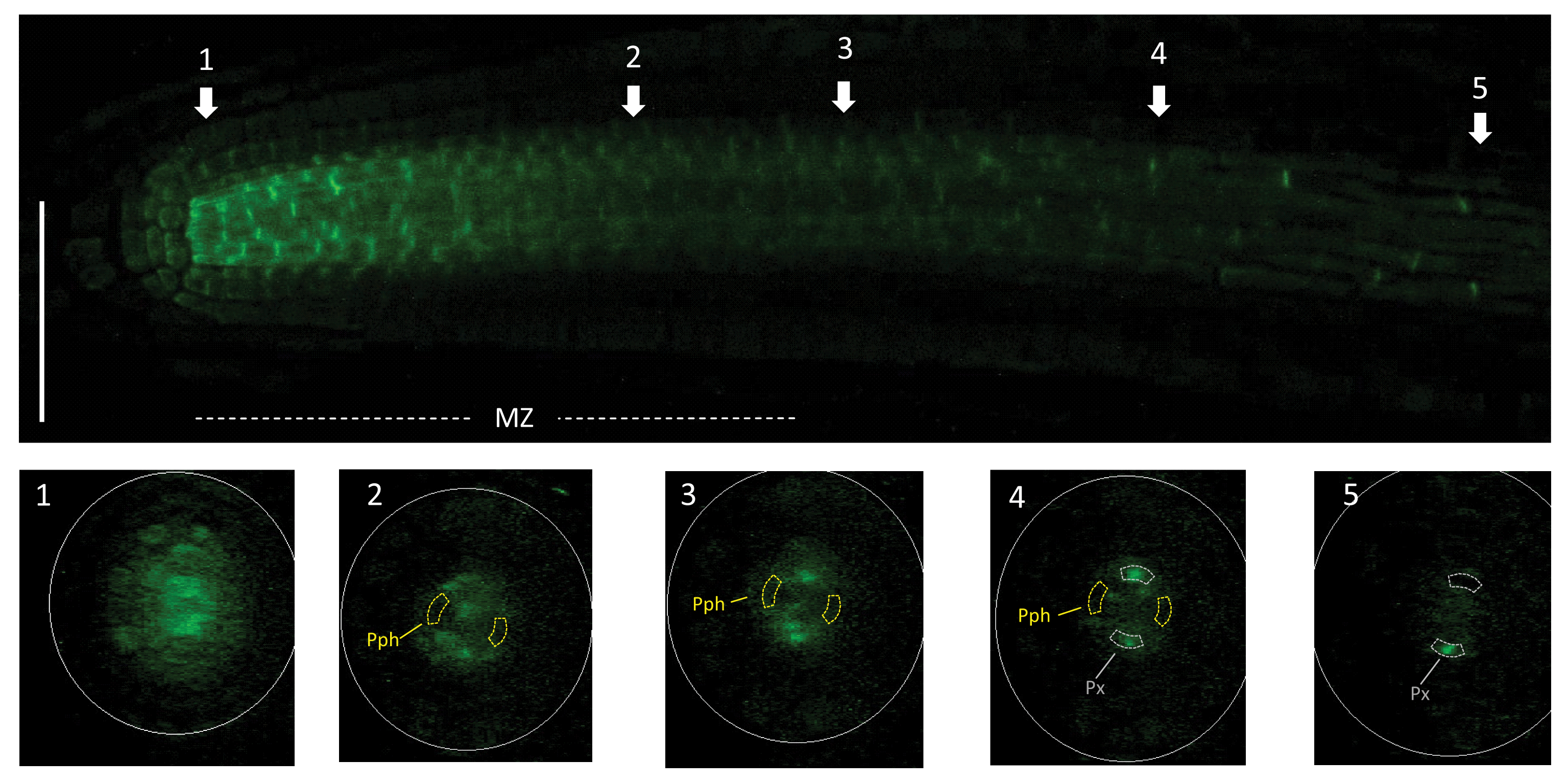

Supplement: Additional file 1: — The details of PIN expression in the root tip, visualized in 3D. The longitudinal section is above. Five transverse section made at different lengths from the QC are shown below. The signal disappears in the upper third of the meristematic zone (MZ) in the protophloem (pph) and protophloem-pole pericycle. In the distal part of elongation zone protoxylem (px), cells still express PIN1. Bars = 50 μm. (TIF 13846 kb) [file 12870_2015_685_MOESM1_ESM.tif]

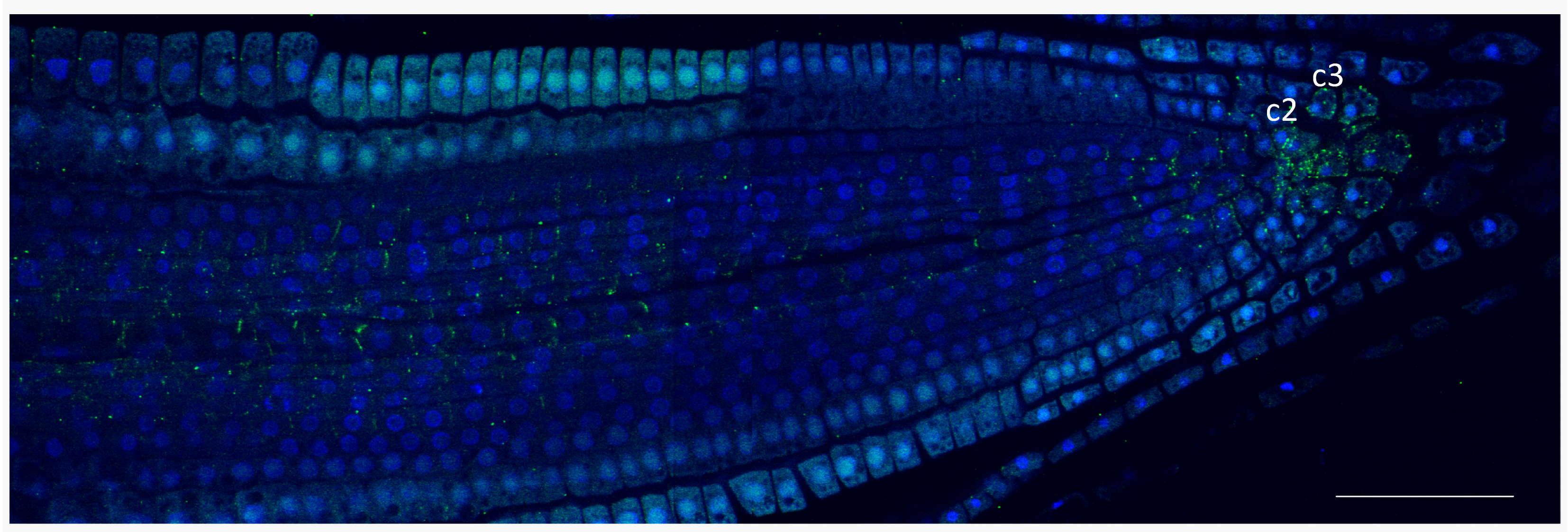

Supplement: Additional file 3: — PIN1 expression pattern in the pin1 mutant (negative control). A weak signal is present in the second and third columella tiers (c2, c3). Anti-PIN1 staining is in green, DAPI is in the blue channel. Bars = 50 μm. (TIF 10907 kb) [file 12870_2015_685_MOESM3_ESM.tif]

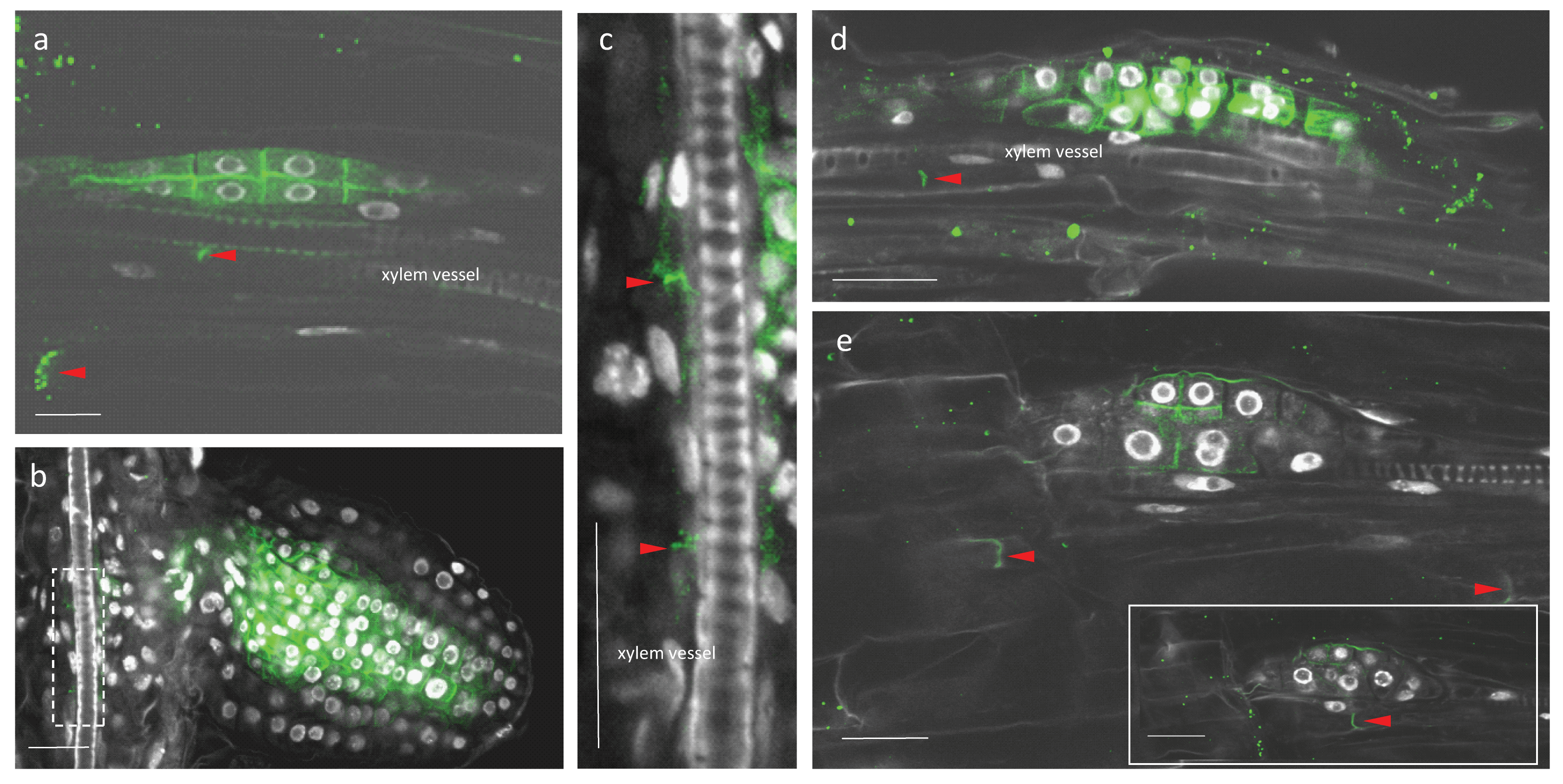

Supplement: Additional file 4: — PIN1 expression in closer proximity to the developing lateral primordium. a-d. PIN1 signal (red arrows) was detected in the xylem elements. c. Magnification of the white box from figure b. e. PIN1 signal (red arrows) was detected in individual cells of the outer layers of the primary root. The white inset shows the same primordium, but with a different focal plane. Anti-PIN1 staining is in green, DAPI is in the white channel. Bars = 50 μm. (TIF 16686 kb) [file 12870_2015_685_MOESM4_ESM.tif]

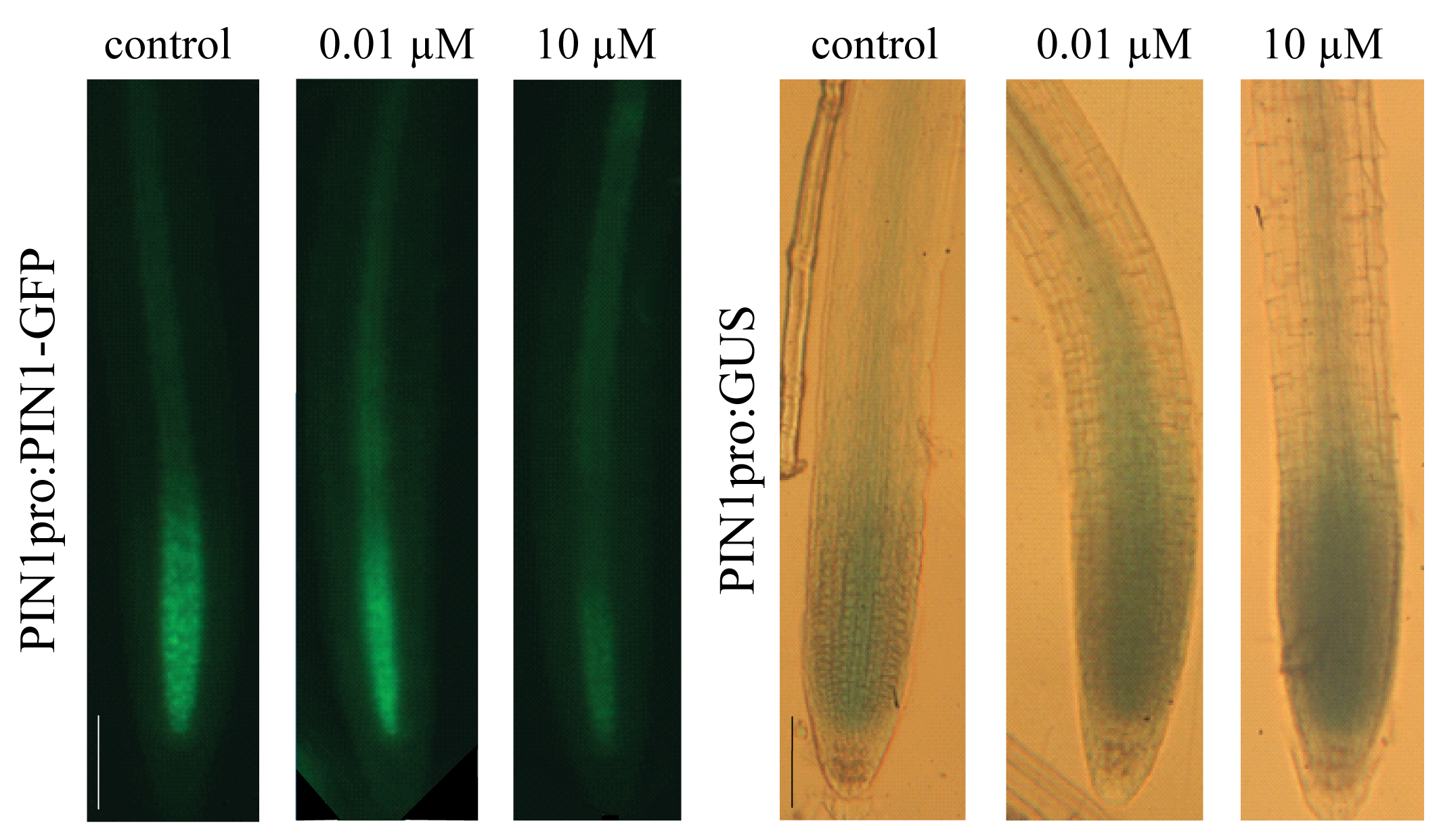

Supplement: Additional file 5: — Auxin treatment effect on the reporter lines PIN1pro:PIN1-GFP and PIN1pro:GUS. Examples of PIN1-GFP expression (a) and GUS staining (b) are given for the roots under low (0.01 μМ IAA) and high (10 μМ IAA) treatments, and these are compared with the control. Bars = 50 μm. (TIF 7755 kb) [file 12870_2015_685_MOESM5_ESM.tif]
