# Supplementary material for: A detailed expression map of the PIN1 auxin transporter in Arabidopsis thaliana root
Source: BMC Plant Biol. 2016 Jan 27;16(Suppl 1):5. doi: 10.1186/s12870-015-0685-0 (PMC4895256; doi:10.1186/s12870-015-0685-0)
Supplement: Additional file 2: — Stable and variable features of the PIN1 expression domain in the meristem. The first column indicates cell position along the central root axis: CSC—columella initials, CEI—cortex/endodermis initials, ELI—epidermis/lateral root cap initials, QC—the quiescent centre. 1–30 - the cell numbers in the lineage from the QC. RW—rootward polarity, NP—nonpolar, RWL—rootward and lateral polarity (on the both sides of the plasma membrane), RWLI—rootward with spreading to the inner lateral side, RWLIp—the same as RWLI, but only part of the inner lateral side is occupied by PIN1. The typical (most frequent) polarity is highlighted in bold font. (DOCX 15 kb) [file 12870_2015_685_MOESM2_ESM.docx]

**Additional file 2.** **Stable and variable features of the PIN1 expression domain in the meristem.**

The first column indicates cell position along the central root axis: CSC – columella initials, CEI – cortex/endodermis initials, ELI- epidermis/lateral root cap initials, QC – the quiescent centre. 1-30 - the cell numbers in the lineage from the QC. RW – rootward polarity, NP – nonpolar, RWL – rootward and lateral polarity (on the both sides of the plasma membrane), RWLI – rootward with spreading to the inner lateral side, RWLIp – the same as RWLI, but only part of the inner lateral side. The typical (most frequent) polarity is highlighted in bold font.

| № of row | epidermis | cortex | endodermis | pericycle | vasculature | protoxylem |
| --- | --- | --- | --- | --- | --- | --- |
| 30 |  |  | RW, RWLIp | RW, RWLI, RWLIp | RW | RW |
| 29 |  |  |  |  |  |  |
| 28 |  |  |  |  |  |  |
| 27 |  |  |  |  |  |  |
| 26 |  |  |  |  | RW, RWL, RWLI |  |
| 25 |  |  |  |  |  |  |
| 24 |  |  |  |  |  | **RW** |
| 23 |  |  |  |  |  |  |
| 22 |  |  | **RW**, RWLIp |  |  |  |
| 21 |  |  |  |  |  |  |
| 20 |  |  |  |  |  |  |
| 19 |  |  |  |  |  |  |
| 18 |  |  |  |  |  |  |
| 17 |  |  |  | **RW,** RWLI, RWLIp | **RW**, RWL, RWLI |  |
| 16 |  | RW |  |  |  |  |
| 15 |  |  |  |  |  |  |
| 14 |  |  |  |  |  |  |
| 13 |  |  |  |  |  |  |
| 12 |  |  |  |  |  | **RW**, RWL, RWLI |
| 11 |  |  |  |  |  |  |
| 10 |  |  | **RW**, RWL, RWLIp |  |  |  |
| 9 |  |  |  | **RW,** RWL, RWLI, RWLIp |  |  |
| 8 |  |  |  |  |  |  |
| 7 | RW, RWL |  |  |  |  |  |
| 6 |  | **RW**, RWL |  |  |  |  |
| 5 |  |  |  |  |  |  |
| 4 |  |  |  |  |  |  |
| 3 |  |  |  |  |  |  |
| 2 | RWL, NP |  |  | **RWL** | **RWL** | **RWL** |
| 1 |  | **RWL** | **RWL** |  |  |  |
| EI/ CEI/ QC | **NP,** RWL | **NP,** RWL | | **NP,** RWL | | |
| CSC | **NP,** RWL | | | | | |
