# Supplementary material for: A detailed expression map of the PIN1 auxin transporter in Arabidopsis thaliana root
Source: BMC Plant Biol. 2016 Jan 27;16(Suppl 1):5. doi: 10.1186/s12870-015-0685-0 (PMC4895256; doi:10.1186/s12870-015-0685-0)
Supplement: Additional file 6: — PIN1 expression in the roots of A. thaliana pin mutants. PIN1 positioning on the plasma membrane was described as follows: RW—rootward, RWLI—rootward and lateral internal, SW—shootward. The percentages of roots having the listed features are shown in brackets. QC—the quiescent centre, CSC—columella stem cells, LRC—the lateral root cap. (DOCX 13 kb) [file 12870_2015_685_MOESM6_ESM.docx]

**Additional file 6.** **PIN1 expression in the roots of *A. thaliana* *pin* mutants.**

PIN1 positioning on the plasma membrane was described as follows: RW – rootward, RWLI – rootward and lateral internal, SW – shootward. The percentages of roots having the listed features are shown in brackets. QC – the quiescent centre, CSC – columella stem cells, LRC – the lateral root cap.

|  | Epidermis | Cortex | Endodermis | Vasculature | QC | CSC | Columella | LRC |
| --- | --- | --- | --- | --- | --- | --- | --- | --- |
| *pin2*  *(15 roots)* | Expression from the 1^st^ to 5th cell row from QC (100%); RW.  Ectopic expression from the 6^th^ to 16th cell row from QC (75%); SW.  From the 17^th^ to 23th cell row, expression gradually decreased. | Expression from the 1^st^ to 18th cell row from QC (100%); RW.  From the 18^th^ to 24th cell row, expression gradually decreased. | Expression from the 1^st^ to 28th cell row from QC (100%); RWLI | Expression from the 1^st^ to 21st cell row from QC (100%); RW. | Expression is present (100%); non polar | Expression is present (100%); non polar | Expression in the second tier (85%); non polar | absent |
| *pin3*  *(5 roots)* | Expression in the 1^st^ to 5th cell row from QC (80%); RW. | Expression in the 1^st^ to 10th cell row from QC (100%); RW.  From 11^th^ to 32th cell row expression gradually decreased. | Expression in 1^st^ to 24th cell rows from QC. (100%); RWLI  From 25^th^ to 47^th^ cell row expression gradually decreased. | Expression in the 1^st^ to 26th cell row from QC (75%); RW. | Expression is present (100%); non polar | Expression is present (100%); non polar | Expression from the 2^nd^ to 5th tier (72%); non polar | absent |
| *pin4*  *(14 roots)* | Expression in the 1^st^ to 5th cell row from QC (100%); RW. | Expression in the 1^st^ to 10th cell row from QC (100%); RW.  From 11^th^ to 20^th^ cell row, expression gradually decreased. | Expression in the 1^st^ to 20th cell row  from QC (100%); RWLI From 21th to 39th cell row, expression gradually decreased. | Expression from the 1^st^ to 31 cell row from QC (70%); RW. | Expression is present (100%); non polar | Expression is present (100%); non polar | Expression in the second tier (14%); non polar | absent |
| *pin7*  *(5 roots)* | Expression in the 1^st^ to 4th cell row from QC, (40%); RW | Expression in the 1^st^ to 5th cell row from QC, (60%); RW. From 6^th^ to 15th cell row, expression gradually decreased. | Expression in the 1^st^ to 20th cell row from QC, (100%); RWLI. From 21th to 29^th^ cell row, expression gradually decreased. | Expression from the 1^st^ –to 34th cell row from QC (60%); RW. | Expression is present (100%); non polar | Expression is present (100%); non polar | Expression in the second tier (80%); non polar | absent |
